# How Body Esteem Influences Virtual Model Selection and Intention to Use Virtual Fitting Rooms

**DOI:** 10.3390/bs15111526

**Published:** 2025-11-10

**Authors:** Ruijuan Wu, Huizhen Jin

**Affiliations:** 1 School of Management, Tianjin University of Technology, Tianjin 300384, China; jennie0212@sina.com; 2 Northeast Asian Research Center, Northeast Asian Studies Collage, Jilin University, Changchun 130012, China

**Keywords:** body esteem, virtual models, virtual fitting rooms, trust, intention to use

## Abstract

Virtual fitting rooms have come a long way, incorporating augmented reality, artificial intelligence, and body scanning technologies to enhance the shopping experience. While the extant literature has provided systematic examinations of consumers’ experience for virtual fitting rooms, factors that affect consumers’ virtual model selection and intention to use virtual fitting rooms remain understudied. This study aims to explore how consumers’ body esteem influences virtual model selection, trust in virtual models, and intention to use virtual fitting rooms. The results of an empirical study in China showed that consumers with high (vs. low) body esteem were more willing to select virtual models with body sizes that were congruent with (vs. larger than) their own, and they were more likely to trust virtual models and use virtual fitting rooms. The preference for thin models and the need for uniqueness produced a moderating effect. These results provide valuable insights into consumers’ intention to use virtual fitting rooms in e-commerce.

## 1. Introduction

In 2024, retail e-commerce sales are estimated to reach USD 4.1 trillion (Statista, 2024). The National Bureau of Statistics of China showed that the number of Chinese online shoppers had reached 1.092 billion and national online retail sales had reached CNY 15.42 trillion by the end of December 2023 (National Bureau of Statistics, 2024). Despite the large number of online shoppers and the large volume of online retail sales, the low conversion rate of consumers from browsers to buyers and the high cart abandonment rate remain two important problems e-retailers are facing. Carmicheal (2021) noted the average cart abandonment rate is 86% globally, which means that one hundred people begin the shopping process on a website, and only fourteen complete the checkout process. The reason for high cart abandonment rates is consumers’ inability to physically examine the products (M. Kim & Lennon, 2008). Consumers cannot touch, smell, or taste the item before purchase, and they feel uncertainty and risk regarding the product’s description and performance (Dimoka et al., 2012). To solve this problem, some e-retailers use augmented reality technology, which decreases consumers’ perceived risk and enhances consumers’ telepresence. Augmented reality (AR) is a technology that superimposes virtual objects onto the physical environment (Tan et al., 2022). This technology integrates virtual information with the user’s real world and allows users to feel realistic virtual AR content (Rauschnabel, 2021). Apparel e-retailers use augmented reality technology to provide services, such as an AR mirror, virtual try-on technology (J. Kim & Forsythe, 2008; Merle et al., 2012), and a virtual fitting room (H. Lee et al., 2020a, 2020b).

In virtual fitting rooms, consumers can virtually try on apparel products with personalized human or virtual models, can select apparel items (such as shirts, dresses, accessories, and shoes) to mix and match from the product catalog, and can see how the outfit looks to entertain themselves (H. Lee et al., 2020a, 2020b; Merle et al., 2012). Virtual fitting rooms provide realistic fitting experiences, decrease the uncertainty of clothing fit, improve the apparel’s trial quality (Gültepe & Güdükbay, 2014), enhance consumers’ utilitarian and hedonic value, and increase consumers’ adoption intentions (H. Lee et al., 2020a, 2020b). According to a report by Grand View Research (2021), the global market size of virtual fitting rooms is expected to reach USD 15.43 billion by 2028. Virtual fitting rooms are welcomed by e-retailers and consumers.

The virtual model is an essential component of virtual fitting rooms. Although technology allows consumers to personalize their own virtual model according to their height, facial features, hair color, and body shape, most virtual fitting rooms provide their own virtual models, including three types of virtual male models (thin, average size, and larger size) and three types of virtual female models (thin, average size, and larger size) (Appendix A). The extant literature has examined the effects of different-sized models. For example, the literature has noted that thin models lead to negative outcomes. Borland and Akram (2007) found that most women cannot sustain the “thin-ideal” body, and the discrepancy between ideal body image and realistic body image elicits dissatisfaction with their own body and image. This dissatisfaction in turn leads to conditions such as low self-esteem (Tiggemann & Lynch, 2001), depressive symptoms (Holsen et al., 2001), anxiety (Halliwell & Dittmar, 2004; Moreno-Domínguez et al., 2018), and dieting behaviors (Markey & Markey, 2005). By contrast, large-sized models (or plus-sized models) lead to more positive outcomes. For example, exposure to large-sized models decreases body dissatisfaction (Moreno-Domínguez et al., 2018), makes consumers feel less pressure from society to be slim (Martin & Xavier, 2010), and encourages women to have more positive thoughts about their own bodies (Peck & Loken, 2004). Shoenberger et al. (2019) found that large-sized models were associated with more positive attitudes and higher purchase intentions than thin models.

Model body size has always been explored as an independent variable and used as a stimulus (or cues) to evaluate people’s responses. Few studies have allowed people to actively choose models. Currently, because of augmented reality technology, apparel e-retailers provide virtual fitting rooms to allow people to make their own choices about virtual models, presenting a good opportunity for us to understand how consumers select virtual models.

Therefore, in the current research, we seek to explore how consumers’ body esteem influences their virtual model selection and their trust in virtual models, which in turn influences their intention to use virtual fitting rooms, and the moderating role of the preference for thin models and need for uniqueness. The following five research questions (i.e., hypotheses) are formulated: (1) Consumer’s body esteem significantly influences their selection of virtual models; (2) body esteem significantly influences on their trust in virtual models; (3) preference for thin models moderates the effect of body esteem on trust in models; (4) trust in virtual models mediates the influence of body esteem on consumer’s intention to use virtual fitting room; (5) need for uniqueness moderates the effect of trust in virtual models on intention to use virtual fitting rooms.

The current research contributes to the emerging research on virtual fitting rooms by focusing on relationships between consumers’ body esteem and virtual model selections, and the findings provide important managerial implications for e-retailers and virtual fitting-room app providers to leverage this new technology smartly to enhance business performance. Specifically, the current research offers several contributions. First, we contribute to the emerging research on virtual fitting rooms by shifting the perspective away from the product and technical level to how the “self” affects the consumers’ intention to use virtual fitting rooms. The extant literature has explored how the virtual fitting room’s overall characteristics, such as interactivity, vividness, and augmentation (H. Lee et al., 2020a, 2020b), influenced consumers’ attitude or intention. The present paper focuses on two antecedents of using virtual fitting rooms, body esteem and trust in virtual models, and also identifies novel moderators (i.e., preference for thin models, need for uniqueness), which highlight the importance of examining virtual fitting rooms from a consumer self-concept perspective and contribute to a more complete understanding of virtual fitting rooms’ impact. Second, we enrich the study of body esteem. While the prior literature has explored the effects of body esteem on consumers’ satisfaction (Merle et al., 2012), anxiety, and comparative behavior (You & Shin, 2019), our work suggests that in VR marketing, consumers’ body esteem can play an essential role in trust and their use intention. Third, we provide new insights into the effects of human model sizes. In previous studies, the body size of a model was considered as an independent variable affecting consumers’ self-perception and attitudes toward advertisements and brands (Martin et al., 2007; Moreno-Domínguez et al., 2018), as well as purchase intentions (Lou & Tse, 2021; Lou et al., 2019). In this study, we consider virtual model selection as a dependent variable, where participants can make their own choices about the different body sizes of virtual models, which allows researchers to obtain a better understanding of consumers’ responses to models.

In the remainder of this article, we first review the literature and develop hypotheses; then, we use an empirical study to test our hypotheses and finally propose theoretical and managerial implications and suggest future research directions.

## 2. Literature Review and Hypothesis Development

### 2.1. Virtual Fitting Rooms and Virtual Models

Virtual fitting rooms were first introduced in 2005 and were widely used and studied. As an emerging virtual reality technology, virtual fitting rooms allow consumers to virtually try on products, integrate apparel products and virtual models, and translate the physical-store fitting experience into an online-store environment (H. Lee et al., 2020a, 2020b). Virtual fitting rooms significantly influence consumers’ online shopping experience because of their interactivity and vividness and their potential for providing an immersive experience (J. H. Kim et al., 2021; H. Lee et al., 2020b). Consumers can use personalized virtual models or virtual models provided by e-retailers to try on clothing. Using virtual fitting rooms to try clothes on decreases product uncertainty before purchase and provides entertainment through mixing and matching items (H. Lee et al., 2020a). Many apparel e-retailers, such as Rebecca Minkoff, Uniqlo, and Timberland, and many other e-retailing platforms, such as www.jd.com (accessed on 5 January 2023) and www.taobao.com (accessed on 7 January 2023), provide virtual fitting rooms to satisfy people’s need to experience fit (H. Lee et al., 2020b).

Virtual models in virtual fitting rooms represent consumers’ body size, facial characteristics, hair color, and other body features (Shim & Lee, 2011). The design of virtual models is based on consumers’ body measurements or data obtained through body scanning machines and camera-based technology (H. Lee & Xu, 2018). Thus, consumers can select virtual models according to their gender, body proportion, height, and body size. Using their selected virtual models, consumers can examine apparel images, view how the apparel looks on the virtual models, and avoid buying products that do not fit (Shim & Lee, 2011). Virtual models have good interactivity, help consumers decrease the perceived risk related to apparel products (Shim & Lee, 2011), and influence consumers’ telepresence and attitudes (H. Lee et al., 2020b). Regarding models’ body size, Lou and Tse (2021) used a nine-point body-size scale: Thin models are below size 3, average-sized models are between size 4 and size 6, and large-sized models are between size 7 and size 9. De Lenne et al. (2021) calculated the following measurements of thin models: a body mass index (BMI) below 20, a bust between 80 and 85 cm, a waist between 60 and 65 cm, and hips between 85 and 88 cm. Dittmar and Howard (2004) noted that thin models were approximately a size 2 (US) or a size 8 (UK) (with a waist circumference of less than 24 inches); average-sized models were approximately a size 12–14 (US) or a size 14 (UK) (with a waist circumference of less than 30 inches). Halliwell and Dittmar (2004) also used US size 12 or UK size 14 to represent the average size. Large-sized models are approximately a size 16–18 (US) (Peck & Loken, 2004) or a US size 16 and above (Bian & Wang, 2015). In this paper, we focus on virtual models’ body size. Generally, virtual fitting rooms provide three kinds of models: thin models, average-sized models, and large-sized models.

### 2.2. Consumer’s Body Esteem and Virtual Model Selection

Body esteem refers to people’s positive or negative feelings toward different aspects of their body and reflects how people like or dislike their body and their appearance (Rosa et al., 2006). In the extant literature, body esteem includes three subdimensions: body esteem–weight (weight satisfaction), body esteem–appearance (appearance satisfaction), and body esteem–attribution (positive attributions about people’s bodies or appearance). Because this paper mainly explores how people evaluate themselves using external rather than internal criteria, we focused on the first two subdimensions and did not use the third subdimension.

Body esteem significantly influences consumers’ evaluation of their own bodies. Heavier children, adolescents, and adults have lower body esteem than do their average-weight peers (Mendelson et al., 2000). Individuals with high (vs. low) body esteem are more confident about and satisfied with their bodies (Merle et al., 2012; You & Shin, 2019). Compared to those high in body esteem, individuals with low body esteem tend to be negatively influenced by an external stimulus or social comparisons, such as a beautiful mannequin or another individual who is attractive, as these draw attention to the perceived deficiencies in their appearance (Argo & Dahl, 2017). Body esteem also significantly influences consumers’ beliefs and purchase intentions. For example, Rosa et al. (2006) found that body esteem has a direct influence on involvement with apparel and has an indirect impact on purchase intention. Bian and Wang (2015) noted that for new brands, individuals with low self-esteem evaluated average models as more attractive than slim models. Merle et al. (2012) pointed out that consumers with high body esteem perceive that they are more congruent with virtual models and are more confident in fit. Self-congruence can also lead to favorable responses, such as brand attachment (Javornik et al., 2021).

When consumers have low body esteem, they tend to evaluate their body as heavier than average weight, and they view their body as unattractive and a negative image, leading to a dislike of their body. At this time, when they choose virtual models in the fitting room, they prefer large-size models because they believe the images of large-size models are not perfect and are congruent with their imagined imperfect images. However, those with high body esteem do not experience such negative effects (Dahl et al., 2011) and prefer models with body sizes that are consistent with their own. Thus, we propose the following hypothesis:

**H1.**  *Consumers’ body esteem significantly influences their selection of virtual models. To be specific, for consumers with high body esteem, they select virtual models with body sizes that are consistent with their own; for consumers with low body esteem, they prefer large-size virtual models.*

### 2.3. Consumer’s Body Esteem and Trust in Virtual Models

In this paper, trust refers to the consumer’s belief in a virtual model’s ability to provide honest and reliable information (Ohanian, 1991; Pickett & Brison, 2019). Consumers believe that a trustworthy virtual model can be relied upon to reflect consumers’ body size. For example, people’s trust in the nonidealized human model is significantly higher than in the idealized human model (Antioco et al., 2012). The reason is that a nonidealized model is a real representation of the world, is similar to the consumer, and is authentic. Pickett and Brison (2019) found that men’s body image inversely influenced the trust of the endorser. Men with a negative body image gave more credence to the former athlete endorser with a negative body image.

In current research, consumers with high body esteem are more satisfied with their own bodies and more confident in their bodies (Merle et al., 2012). They downplay the unsatisfactory aspects of their bodies to maintain high body esteem and make themselves feel good (Powell et al., 2001). Consumers with high body esteem will more likely select virtual models with body sizes that are consistent with their own. Thus, they will have positive attitudes toward virtual models that are consistent with their own and have more trust in them. By contrast, low body esteem is associated with body dissatisfaction, increased body anxiety, engaging in more comparative behavior, and stronger internalization of the thin ideal (You & Shin, 2019). Thus, they tend to select imperfect large-sized virtual models and believe that the models can represent their body, increasing their trust in those virtual models.

Therefore, we propose the following:

**H2.**  *Body esteem significantly influences consumers’ trust in virtual models. To be specific, for consumers with high body esteem, they are more likely to trust a virtual model with a body size that is consistent with their own; for consumers with low body esteem, they are more likely to trust a large-sized virtual model.*

### 2.4. Consumer’s Body Esteem, Preference for Thin Models, and Trust in Virtual Models

Consumers like different body size models. Some consumers prefer thin models because thin models are perceived to be more attractive (Halliwell & Dittmar, 2004). The media and family members support people’s preference for thin models, as indicated by comments criticizing overweight bodies and encouraging dieting behavior (Borland & Akram, 2007). A thin body is regarded as the ideal body type and has dominated fashion for many years, and consumers believe that thin-ideal models are common in e-commerce marketing because clothes hang and drape better on them (Aagerup & Scharf, 2018). Martin et al. (2007) found that, for people who believe that they can control their weight, thin models lead to more favorable attitudes toward advertisements, brand attitudes, and purchase intentions.

In the current research, we propose that preference for thin models moderates the effect of consumers’ body esteem on trust in virtual models. Some consumers have the belief that models should be thin, and thinness is desirable and positive (Ketelaar et al., 2014). Clothes in virtual fitting rooms look better on thin models, and these consumers prefer thin models. For these consumers, the effect of body esteem on trust in models will decrease. In other words, consumers with low body esteem who prefer thin models and those who are not negatively influenced by thin (or average-sized and large-sized) models tend to have increased trust in virtual models, similar to high body esteem consumers. However, when consumers have a weak preference for thin models, the difference in the effect of body esteem on trust in models remains unchanged. Thus, we proposed the following:

**H3.**  *Preference for thin models moderates the effect of body esteem on trust in models. Specially, for consumers who have a strong preference for thin models, the difference between high body esteem and low body esteem on trust in virtual models will be attenuated; for consumers who have a weak preference for thin models, the different effect between high body esteem and low body esteem on trust in virtual models remains unchanged.*

### 2.5. Consumer’s Body Esteem, Trust in Virtual Models, and Consumer’s Intention to Use Virtual Fitting Rooms

Even though several of the constructs included in the Technology Acceptance Model (TAM) and Unified Theory of Acceptance and Use of Technology (UTAT) are predictors of users’ intention to use AI technology (Gursoy et al., 2019; Venkatesh et al., 2003; Venkatesh et al., 2012), some of the factors of TAM and UTAT, such perceived usefulness and performance expectancy, heavily depend on consumers’ trust toward AI technology, as shown in several studies that examined determinants of acceptance levels of AI devices (Ozturk et al., 2016; Park, 2020; Tussyadiah et al., 2020). Trust is an essential element in accepting new technology because it can help people overcome their uncertainty with respect to technological advancement (Meyer-Waarden & Cloarec, 2022). It can be defined as “the attitude that an agent will help achieve an individual’s goals in a situation characterised by uncertainty and vulnerability” (J. D. Lee & See, 2004). Trust has been discovered as a vital factor in determining human–automation interaction (Zhang et al., 2019). Thus, trust in virtual models should also be considered as an important determinant that can influence the intention to use virtual fitting rooms. In the same vein, self-images, such as body esteem, play a critical role in perceived trust of virtual models, which is also likely to influence consumers’ intention to use virtual fitting rooms (Yang & Xiong, 2019).

The use of VR technology reduces customers’ perceived risks while increasing trust, and importantly, the interaction between buyer and seller is likely to increase (Allimamy et al., 2019). Virtual fitting rooms offer an interactive function that allows consumers to employ virtual models to try clothes on (Yang et al., 2023). Consumers’ interactive and immersive experience in virtual fitting rooms is mainly dependent on interaction with virtual models. Using virtual models, consumers can evaluate apparel fit, enhancing their confidence in selecting apparel products and telepresence (H. Lee et al., 2020b). Thus, consumers’ trust in virtual models will directly affect their intention to use virtual fitting rooms. The extant literature has tested the relationship between trust and intention to use. For example, such tools have significant and positive effects on consumers’ intention to try on and visualize products (Ganapathy et al., 2004); the power of retention of a website (Häubl & Figueroa, 2002); trust in a product (Trifts & Häubl, 2003); purchase intention (Li et al., 2002); and satisfaction (Murray & Häubl, 2008). Thus, when consumers trust virtual models, their intention to use virtual fitting rooms is enhanced. We propose the following:

**H4.**  *Trust in virtual models mediates the influence of body esteem on consumers’ intention to use virtual fitting rooms.*

### 2.6. Trust in Virtual Models, Need for Uniqueness, and Consumer’s Intention to Use Virtual Fitting Room

Need for uniqueness means “an individual’s pursuit of differentness relative to others” (Tian et al., 2001, p. 50). Consumers with a high need for uniqueness have a strong need to be different (Imhoff & Lamberty, 2017) and fear of becoming common (Cheema & Kaikati, 2010). They dislike following the crowd and have a strong desire to deviate from others (Sharifi, 2020). Avoidance of similarity is one of the behavioral manifestations of the need for uniqueness (Tian et al., 2001). Avoidance of similarity means consumers devalue products or brands that are commonplace. Thus, uniqueness seekers lose interest in, become reluctant to use, or discontinue using these products or brands.

In this paper, we propose that trust in virtual models significantly influences consumers’ usage intention of virtual fitting rooms. However, this influence may be moderated by the consumer’s need for uniqueness. Consumers seeking uniqueness may trust virtual models, but their desire to seek uniqueness makes them dislike virtual models that are provided by virtual fitting rooms and used by most people. They desire other novel interactions rather than interactions with a limited number of virtual models provided by virtual fitting rooms. Thus, they reduce the use of virtual fitting rooms if they believe virtual models are welcomed by many people. Regarding consumers with a low need for uniqueness, these individuals are seeking conformity and are more willing to conform with others (Sharifi, 2020). For those with a low need for uniqueness, when they trust virtual models, they are prone to using virtual fitting rooms. Thus, the enhancement effect of trust in virtual models on the intention to use virtual fitting rooms is still significant. We propose the following hypothesis:

**H5.**  *The need for uniqueness moderates the effect of trust in virtual models on intention to use virtual fitting rooms. Specifically, for consumers with a high need for uniqueness, the enhancement effect of trust in virtual models on intention to use virtual fitting rooms will be attenuated; for consumers with a low need for uniqueness, the enhancement effect remains unchanged.*

**Figure 1:**
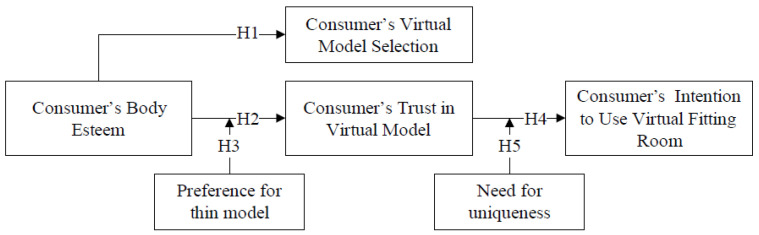
The conceptual framework.

Figure 1 depicts the proposed conceptual framework for the current research.

## 3. Methods

We conducted one empirical study to test all five hypotheses: (1) how consumers select virtual models in virtual fitting rooms according to their own body esteem (H1); (2) the effect of body esteem on trust in virtual models (H2); (3) the moderating role of the preference for thin models (H3); (4) the mediating role of trust in virtual models (H4); and (5) the moderating role of need for uniqueness (H5).

### 3.1. Design and Procedures

To earn course credit, we recruited 482 students (270 females and 212 males) from a major university in China to participate in this study. We used the real “virtual fitting room” app. To exclude the effect of brands, we invented the brand “Yi Shang”.

We first instructed students to download the app and then asked them to read the app’s content: “The brand (Yi Shang) provides the virtual fitting room (see Appendix A) for shoppers. You can freely use models, clothes, accessories, and hair to mix and match.” Next, participants would see six virtual models (three virtual female models: a thin model, an average-sized model, and a large-sized model; three virtual male models: a thin model, an average-sized model, and a large-sized model). We added notes for the virtual models: According to the calculation method of standard weight (height [cm] minus 105) of the World Health Organization, the (1) #1 model (female) and #4 model (male) were thin models for which their body weight was below 90% of the standard weight; (2) the #2 model (female) and #5 model (male) were average-sized models for which their body weight ranged from 90% of the standard weight to 110% of the standard weight; and (3) the #3 model (female) and #6 model (male) were large-sized models for which their body weight was more than 110% of the standard weight. Except for the body size, the other parts (hair, face, and background) of the female model (or the male model) were the same. After reading the content, participants filled in the questionnaire. The first question asked participants to answer which model they would like to select. Participants selected one virtual model (see Appendix B). We positioned virtual model selection as the first question to measure the direct response of participants when they were exposed to different body-size models. Further, we presented six models at the same time to avoid upward or downward comparisons between the virtual model and the self. Then, participants answered questions regarding measurements of trust in virtual models, preference for thin models, body esteem, need for uniqueness, and intention to use virtual fitting rooms. Finally, participants evaluated their own body size and reported their gender.

Of the 482 received questionnaires, except for incomplete questionnaires (4 questionnaires) and questionnaires not answered seriously (46 questionnaires, in which male participants selected female models and female participants selected male models that are not valid in this study in terms of gender incongruence), 432 questionnaires (245 female and 187 male) were valid, and the data analysis was based on the 432 valid questionnaires.

### 3.2. Measures

We adopted scales from the literature regarding five variables in this paper, and these five variables were measured using seven-point Likert scales (1 = totally disagree; 7 = totally agree). Trust in virtual models (α = 0.888) was measured using four items adopted from Antioco et al. (2012): for example, “I think the virtual model I selected honestly reflects my body”. Preference for thin models (α = 0.912) was adapted from Ketelaar et al. (2014) and was measured using five items: for example, “I prefer seeing thin models”. Body esteem (α = 0.910) was measured using five items adopted from Merle et al. (2012): for example, “I feel satisfied with the way my body looks right now”. Four items were used to measure need for uniqueness (α = 0.808): for example, “I prefer being different from other people”. This measure was adopted from Lindsey-Hall et al. (2021). Three items were used to measure intention to use virtual fitting rooms (α = 0.864): for example, “I will frequently use virtual fitting rooms in the future”. This measure was adopted from Hsu et al. (2007).

At the end of the questionnaire, participants evaluated their own body size and reported their gender. The evaluation method for participants’ body size was the same as the calculation method for virtual models. We adopted the body weight calculation method of the World Health Organization: a person’s standard weight equals height (cm)—105. Average size refers to when people’s weight ranges from 90% of the standard weight to 110% of the standard weight; thin size is when people’s weight is below 90% of the standard weight; large size is when people’s weight is above 110% of the standard weight.

## 4. Results

### 4.1. Measurement Property Assessment and Common Method Variance

We employed SPSS 24.0 to conduct exploratory factor analyses. The results showed that all variables’ reliability was strong, with all Cronbach’s α coefficients above 0.80 (Hair et al., 2012).

Further, we employed LISREL 8.70 to conduct a confirmatory factor analysis (CFA) to analyze convergent validity, discriminant validity, and the model’s fit. The result showed that all standardized factor loadings were greater than 0.65, composite reliability (CR) was above 0.80, and averaged variance extracted (AVE) was greater than 0.60, which means that the scales exhibited strong convergent validity (Bagozzi & Yi, 1988; Hair et al., 2012). The scales also exhibited evident discriminant validity because the values of the square root of the AVE were greater than the correlations with other constructs (Fornell & Larcker, 1981). The results of CFA showed that the hypothesized five-factor model and the data demonstrated that goodness-of-fit was adequate (*χ*^2^ = 728.88; df = 179; *χ*^2^/df = 4.07; CFI = 0.95; TLI = 0.94; GFI = 0.95; RMSEA = 0.031; SRMR = 0.049) (see Table 1).

Furthermore, we tested the common method’s variance. According to the study of Podsakoff et al. (2003), we compared the fit between the five-factor model and the data, as well as the fit between the one-factor model and the data. The results showed that the fit between the five-factor model and the data (*χ*^2^/df = 4.07; CFI = 0.95; TLI = 0.94; GFI = 0.95; RMSEA = 0.031; SRMR = 0.049) was significantly better than that between the one-factor model and the data (*χ*^2^/df = 26.57; CFI = 0.56; TLI = 0.52; GFI = 0.40; RMSEA = 0.28; SRMR = 0.21). This indicated that there was no significant common method bias (see Table 2).

For checking the biases in the self-report responses, common method biases (CMBs) were used with Harman’s single-factor test to check the explanation of variance extracted by the individual factor. The findings of the CMB ensure that the variance explained by the individual factor is 31%, which is not greater than 50%, ensuring that the research data did not suffer from bias issues (Podsakoff et al., 2003).

### 4.2. Consumer’s Body Esteem and Virtual Model Selection

We employed Pearson’s chi-squared test to test the relationship between consumers’ body esteem and virtual model selection; to be specific, for consumers with high body esteem, they select a virtual model with a body size that is consistent with their own; for consumers with low body esteem, they prefer large-size virtual models. The current research used consumers’ body esteem as an independent variable, and body esteem was a continuous variable. We divided body esteem into three groups: high body esteem (the mean score + 1SD), medium esteem (between the mean score − 1SD and the mean score + 1SD), and low esteem (the mean score − 1SD). Regarding dependent variables, we recoded the data. If participants selected virtual models with a body size that is congruent with their own body size, we recoded this as 1; if participants selected virtual models with a body size that is thinner than their own, we recoded this as 2; if participants selected virtual models with a body size that is larger than their own, we recoded it as 3. The selection outcome was as follows (see Table 3).

The results showed that for participants with high body esteem, the number of participants who selected virtual models with a body size that was congruent with their own (*n* = 37) was greater than those who selected virtual models with a body size that was larger than their own (*n* = 5) (Wald = 17.65, *p* < 0.001), and it was greater than those who selected virtual models with a body size that was thinner than their own (*n* = 20) (Wald = 4.91, *p* = 0.027). For participants with low body esteem, the number of participants who selected virtual models with a body size that was larger than their own (*n* = 45) was greater than those who selected virtual models with a body size that was congruent with their own (*n* = 15) (Wald = 13.58, *p* < 0.001), and it was greater than those who selected virtual models with a body size that was thinner than their own (*n* = 5) (Wald = 21.73, *p* < 0.001). Thus, H1 is supported. Further, we analyzed participants with medium body esteem, and we found that the number of participants who selected virtual models with a body size that was congruent with their own (*n* = 192) was greater than those who selected virtual models with a body size that was thinner than their own (*n* = 53) (Wald = 68.82, *p* < 0.001), and it was greater than those who selected virtual models with a body size that was larger than their own (*n* = 60) (Wald = 61.85, *p* < 0.001).

### 4.3. Consumer’s Body Esteem, Preference for Thin Models, and Trust in Virtual Models

To test the effect of consumers’ body esteem on trust in virtual models and the moderating effect of preference for thin models, we conducted Bootstrapping Model 1 using the PROCESS macro in SPSS, with 5000 bootstrap samples (Preacher & Hayes, 2004; Preacher et al., 2007). In this analysis, we entered body esteem (X) as the independent variable, preference for thin models (W) as the moderator, and trust in virtual models as the dependent variable (Y). When conducting bootstrapping analysis, body esteem, preference for thin models, and trust in virtual models were all continuous variables and mean-centered.

**Figure 2:**
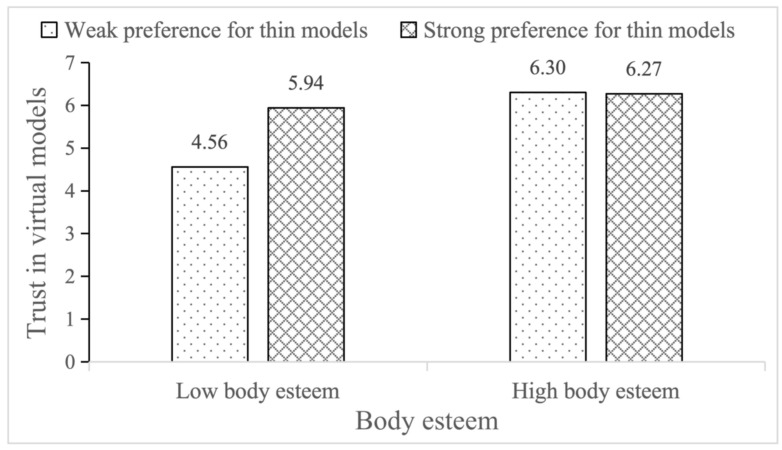
Interaction effects of body esteem and preference for thin models on trust in virtual models.

The bootstrapping analysis results showed that the R^2^ of the model is 0.17 (*p* < 0.001). The main effect of body esteem on trust in virtual models was significant (*β* = 0.80, *t* = 4.90, *p* < 0.001, 95% *CI* = 0.4771, 1.1181) (H2 was supported); the main effect of preference for thin models on trust in virtual models was significant (*β* = 0.51, *t* = 3.35, *p* = 0.001, 95% *CI* = 0.2094, 0.8035); the interaction effects of body esteem and preference for thin models were significant (*β* = −0.09, *t* = −2.97, *p* = 0.003, 95% *CI* = −0.1509, −0.0307). For consumers who had a weak preference for thin models, the effect of body esteem on trust in virtual models was significant (Effect = 0.45, *t* = 7.95, *p* < 0.001, 95% *CI* = 0.3363, 0.5571). For consumers who had strong preference for thin models, the effect of body esteem on trust in virtual models was significant too (Effect = 0.24, *t* = 4.99, *p* < 0.001, 95% *CI* = 0.1477, 0.3397). However, for consumers who had a strong preference for thin models, the enhancement effect of body esteem decreased, and H3 was supported. Figure 2 depicts the interaction effects of body esteem and preference for thin models on trust in virtual models.

Further, we conducted a moderated mediation analysis using PROCESS 3.4, with 5000 bootstrap samples (Model 58). In this analysis, we entered body esteem (X) as the independent variable, preference for thin models (W) as the moderator, trust in virtual models as the mediator (M), and intention to use virtual fitting rooms as the dependent variable (Y). We note that body esteem, preference for thin models, trust in virtual models, and intention to use virtual fitting rooms are all continuous variables and mean-centered. The results showed that the indirect effect of the interaction on the intention to use virtual fitting rooms through trust in virtual models was significant. Specifically, the conditional indirect effect of body esteem excluded zero in both the strong preference for thin models condition (*β* = 0.52, 95% *CI* = 0.4260, 0.6089) and in the weak preference for thin models condition (*β* = 0.84, 95% *CI* = 0.7560, 0.9321).

### 4.4. The Mediating Role of Trust in Virtual Models

According to the bootstrapping procedure proposed by Preacher and Hayes (2004) and Preacher et al. (2007), we added trust in the virtual model and used 5000 bootstrap samples (model 4) to test the mediating role of trust in virtual models. The bootstrapping analysis’s results showed that the R^2^ of the model is 0.49 (*p* < 0.001). The significant effect of body esteem on intention to use virtual fitting rooms was insignificant (*β* = 0.126, *t* = 1.86, *p* = 0.06, 95% *CI* = −0.0074, 0.2590), and trust in virtual models significantly influenced the intention to use virtual fitting rooms (*β* = 0.692, *t* = 18.99, *p* < 0.001, 95% *CI* = 0.6205, 0.7638). Trust in virtual models fully mediated the effect of body esteem on intention to use virtual fitting rooms (Effect = 0.39, 95% *CI* = 0.2626, 0.5412). Thus, H4 was supported.

### 4.5. Trust in Virtual Models, Need for Uniqueness, and Consumers’ Intention to Use Virtual Fitting Rooms

To test the moderating effect of need for uniqueness between trust in virtual models and consumers’ intention to use virtual fitting rooms, we conducted Bootstrapping Model 1 using PROCESS, with 5000 bootstrap samples. In this analysis, we entered trust in virtual models (X) as the independent variable, need for uniqueness (W) as the moderator, and consumer’s intention to use virtual fitting rooms as the dependent variable (Y). When conducting bootstrapping analysis, trust in virtual models, need for uniqueness, and consumers’ intention to use virtual fitting rooms were all continuous variables and mean-centered.

**Figure 3:**
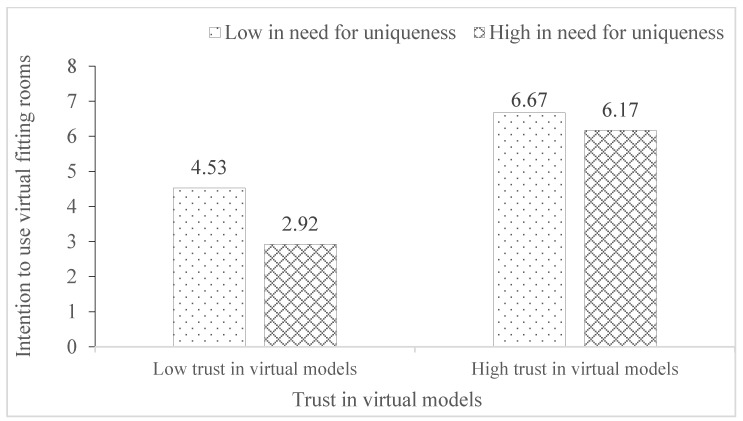
Interaction effects of trust in virtual models and need for uniqueness on intention to use virtual fitting rooms.

The bootstrapping analysis results showed that the R^2^ of the model is 0.55 (*p* < 0.001). The main effect of trust in virtual models on intention to use virtual fitting rooms was significant (*β* = 0.54, *t* = 3.85, *p* < 0.001, 95% *CI* = 0.2669, 0.8227), the main effect of need for uniqueness on intention to use virtual fitting rooms was significant (*β* = −0.53, *t* = −3.17, *p* = 0.002, 95% *CI* = −0.8568, −0.2016), and the interaction effects of trust in virtual models and need for uniqueness were marginally significant (*β* = 0.06, *t* = 1.82, *p* = 0.069, 95% *CI* = −0.0044, 0.1168). For participants low in need for uniqueness, the effect of trust in virtual models on intention to use virtual fitting rooms was significant (Effect = 0.76, *t* = 18.38, *p* < 0.001, 95% *CI* = 0.6745, 0.8360); for participants high in need for uniqueness, the effect of trust in virtual models on intention to use virtual fitting rooms was significant (Effect = 0.87, *t* = 15.70, *p* < 0.001, 95% *CI* = 0.7636, 0.9821). However, for participants high in need for uniqueness, the enhancement effect of trust in virtual models was not attenuated, and H5 was partially supported. Figure 3 depicts the interaction effects of trust in virtual models and need for uniqueness on intention to use virtual fitting rooms.

Table 4 presents a summary of the hypotheses supported.

## 5. Conclusions and Discussion

### 5.1. Conclusions

The present paper explores how consumers’ body esteem influences consumers’ virtual model selection, trust in virtual models, and intention to use virtual fitting rooms. The results showed that body esteem significantly influenced consumers’ virtual model selection and trust in virtual models. Regarding consumers with high body esteem, they are more willing to select virtual models with a body size that is congruent with their own, and they are more likely to trust virtual models; for consumers with low body esteem, they are more willing to select virtual models with a body size is larger than their own (H1 was supported), and they are more likely to trust virtual models (H2 was supported). Trust in virtual models mediated the effect of body esteem on intention to use virtual fitting rooms (H4 was supported). This finding indicates that virtual models are crucial elements in virtual fitting rooms, and they determine consumers’ trying-on experience (Shim & Lee, 2011).

In relationships between body esteem and trust in virtual models, preference for thin models played the moderating role (H3 was supported). For consumers who have a strong preference for thin models, the enhancement effect of body esteem on trust in virtual models decreased. While for consumers who have a weak preference for thin models, the different effect between high body esteem and low body esteem on trust in virtual models remains unchanged. The need for uniqueness moderated the effect of trust in virtual models on the intention to use virtual fitting rooms (H5 was partially supported). Regarding consumers with a high need for uniqueness, when they had less trust in virtual models, their intention to use virtual fitting rooms was extremely weak. Thus, for these consumers, the effect of trust in virtual models on intention to use virtual fitting rooms was enhanced rather than decreased.

### 5.2. Implications

The current research has straightforward and substantial implications for research on model size in the E-commerce field; it enriches the study of the effects of body esteem and research on virtual fitting rooms, especially with respect to the intention to use virtual fitting rooms.

First, the literature has explored the effects of human model size. For example, thin human models elicited favorable attitudes toward advertisements and brands (Martin et al., 2007), but they also resulted in dissatisfaction with consumers’ image and body (Borland & Akram, 2007), lower self-esteem (Tiggemann & Lynch, 2001), and anxiety (Moreno-Domínguez et al., 2018). Average-sized human models enhanced purchase intentions (Lou & Tse, 2021; Lou et al., 2019). Moreover, large-sized human models decreased body dissatisfaction (Moreno-Domínguez et al., 2018) and decreased the pressure to be thin (Martin & Xavier, 2010). In these studies, the model was researched as an independent variable, while in the current paper, participants could make their own choices about virtual models. We used virtual model selection as the dependent variable, and it was helpful for us to understand consumers’ responses to models.

Second, this paper enriches the study of the effects of body esteem. The literature has found that body esteem is related to outcomes such as satisfaction (Merle et al., 2012), anxiety, and comparative behavior (You & Shin, 2019). In the current research, we found that body esteem significantly influences virtual model selection, trust in virtual models, and intention to use virtual fitting rooms. Further, previous research showed that short-term exposure to an image of a thin body (versus a heavy body) resulted in low-esteem women selecting a thinner personal body ideal, and personal body ideals also shifted toward a thinner ideal in the context of thin (versus heavy) body cues (Bair et al., 2014). However, we found that the context of thin body cues was also affected by consumers’ preference for thin models, decreasing the effect of consumers’ body esteem on trust in models, which provides new insights into body esteem research.

Third, this study enriches the research on virtual fitting rooms, especially with respect to the intention to use virtual fitting rooms. The extant literature has explored how the virtual fitting room’s overall characteristics, such as interactivity, vividness, and augmentation (H. Lee et al., 2020a, 2020b), influenced consumers’ attitude or intention. The present paper focused on two antecedents of using virtual fitting rooms: body esteem and trust in virtual models. We also identify novel moderators (i.e., preference for thin model, need for uniqueness), which highlight the importance of examining virtual fitting rooms from a consumer preference perspective and contribute to a more complete understanding of virtual fitting rooms’ impact.

This paper also provides some important managerial implications for e-retailers and virtual fitting-room app providers and offers guidance to leverage this new technology smartly to enhance business performance. Virtual models are an essential factor in virtual fitting rooms, and they are important for consumers’ virtual try-on experience. Body esteem directly influences virtual model selection and trust in virtual models, and it indirectly influences consumers’ intention to use virtual fitting rooms.

In general, with respect to participants’ trust in virtual models, the mean score is 5.18. Thus, e-retailers should pay extra attention to virtual models, which are crucial for consumers’ virtual try-on experiences. Consumers with low body esteem prefer large-size models, and some people have strong preferences for thin models, which decreases the enhancement effect of body esteem on trust in virtual models. Thus, e-retailers can provide different kinds of virtual models, such as virtual models of different sizes, 2D and 3D virtual models, and personalized virtual models. Because body esteem influences consumers’ trust in virtual models, e-retailers can improve consumers’ body esteem. For example, e-retailers can design a system that provides positive feedback (such as praise and encouraging words) when consumers use virtual fitting rooms.

Overall, we encourage managers to acknowledge that consumers respond differently to the same virtual model features and that more resources should be deployed into enabling more customized virtual models.

### 5.3. Limitations and Future Directions

This paper presents some interesting findings, but we acknowledge some limitations. In this paper, we used three body sizes: thin, average-sized, and large-sized. In future research, it would be more interesting to use more body sizes, such as obese sizes and zero size. We used the calculation method of the standard weight, height (cm) minus 105, to determine body size. Although this method is easily calculated and widely used, it may classify muscular individuals as large. Future research can use other calculation methods to determine body size.

Our study is lab-based, and we instructed participants to download a virtual fitting room app to measure their response, which was highly controlled. While this allows for high internal validity, future field and in-the-wild studies (Rogers & Marshall, 2017) could provide greater external validity by examining real-world virtual fitting room interactions. Also, it is more desirable to measure consumers’ responses when they make a real purchase using virtual fitting rooms. Specifically, future research can adopt a longitudinal research design to dynamically track the behavioral changes of consumers after long-term exposure to virtual fitting rooms so as to more accurately assess the long-term effectiveness of virtual fitting rooms. Meanwhile, by combining actual consumption data and market sales data, the correlation between research conclusions and the real market could be further verified, and more reliable data support could be provided for retailers’ marketing decisions.

Moreover, the virtual models used in our study are mainly local Chinese virtual models, and we focused on the body sizes of virtual models. Future research can explore different virtual model ethnicities, styles, and body shapes (pear-shaped, apple-shaped, hourglass-shaped, and so on).

## Figures and Tables

**Table 1:** Results of reliability and convergent validity.

Factors	Items	Factor Loadings (T Value)	Cronbach’s α	CR	AVE
Body Esteem	BE1	0.80 (19.63)	0.910	0.912	0.675
	BE2	0.81 (19.75)			
	BE3	0.88 (22.56)			
	BE4	0.87 (22.12)			
	BE5	0.75 (17.79)			
Preference for thin models	PR1	0.82 (20.15)	0.912	0.912	0.676
	PR2	0.84 (21.01)			
	PR3	0.79 (19.14)			
	PR4	0.81 (20.03)			
	PR5	0.85 (21.25)			
Trust in virtual models	TR1	0.79 (19.06)	0.888	0.892	0.676
	TR2	0.77 (18.37)			
	TR3	0.89 (22.82)			
	TR4	0.84 (21.05)			
Need for uniqueness	NE1	0.66 (16.63)	0.808	0.858	0.605
	NE2	0.87 (21.50)			
	NE3	0.71 (17.49)			
	NE4	0.87 (21.37)			
Intention to use virtual fitting rooms	IN1	0.82 (19.88)	0.864	0.862	0.677
	IN2	0.84 (20.56)			
	IN3	0.81 (19.69)			

Goodness-of-fit indices: *χ*^2^ = 728.88; df = 179, *χ*^2^/df = 4.07; CFI = 0.95; TLI = 0.94; GFI = 0.95; RMSEA = 0.031; SRMR = 0.049.

**Table 2:** Results of discriminant validity.

	BE	PR	TR	NE	IN
BE	**0.821**				
PR	0.11	**0.822**			
TR	0.41	0.12	**0.822**		
NE	0.30	0.49	0.30	**0.778**	
IN	0.33	0.18	0.67	0.06	**0.823**

Note: Numbers in bold are the values of the square root of the AVE; other numbers are the correlations between variables.

**Table 3:** Crosstabulation of participant body size and congruency.

	Congruency		
Participant Body Esteem	Participants Selected Virtual Models with a Body Size That Was Congruent with Their Own Body Size	Participants Selected Virtual Models with a Body Size That Was Thinner Than Their Own	Participants Selected Virtual Models with a Body Size That Was Larger Than Their Own
High	37	20	5
Medium	192	53	60
Low	15	5	45

**Table 4:** Summary of hypotheses supported.

Hypotheses	Result
H1: Consumer’s body esteem significantly influences their selection of virtual models. To be specific, for consumers with high body esteem, they select a virtual model with a body size that is consistent with their own; for consumers with low body esteem, they prefer large-size virtual models.	Supported
H2: Body esteem significantly influences on their trust in virtual models. To be specific, for consumers with high body esteem, they are more likely to trust a virtual model with a body size that is consistent with their own; for consumers with low body esteem, they are more likely to trust a large-sized virtual model.	Supported
H3: Preference for thin models moderates the effect of body esteem on trust in models. Specifically, for consumers who have strong preference for thin models, the difference between high body esteem and low body esteem on trust in virtual models will be attenuated; for consumers who have weak preference for thin models, the different effect between high body esteem and low body esteem on trust in virtual models remains unchanged.	Supported
H4: Trust in virtual models mediates the influence of body esteem on consumer’s intention to use virtual fitting rooms.	
H5: Need for uniqueness moderates the effect of trust in virtual models on intention to use virtual fitting rooms. Specially, for consumers high in need for uniqueness, the enhancement effect of trust in virtual models on intention to use virtual fitting rooms will be attenuated; for consumers low in need for uniqueness, the enhancement effect remains unchanged.	Partially supported, for consumers high in need for uniqueness, the enhancement effect of trust in virtual models was not attenuated.

**Figure A1:**
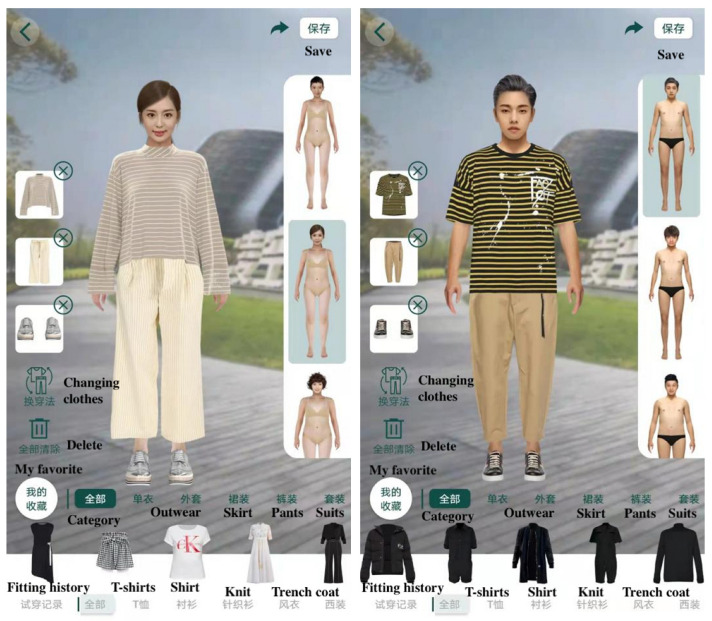
Two web pages in virtual fitting rooms.

**Figure A2:**
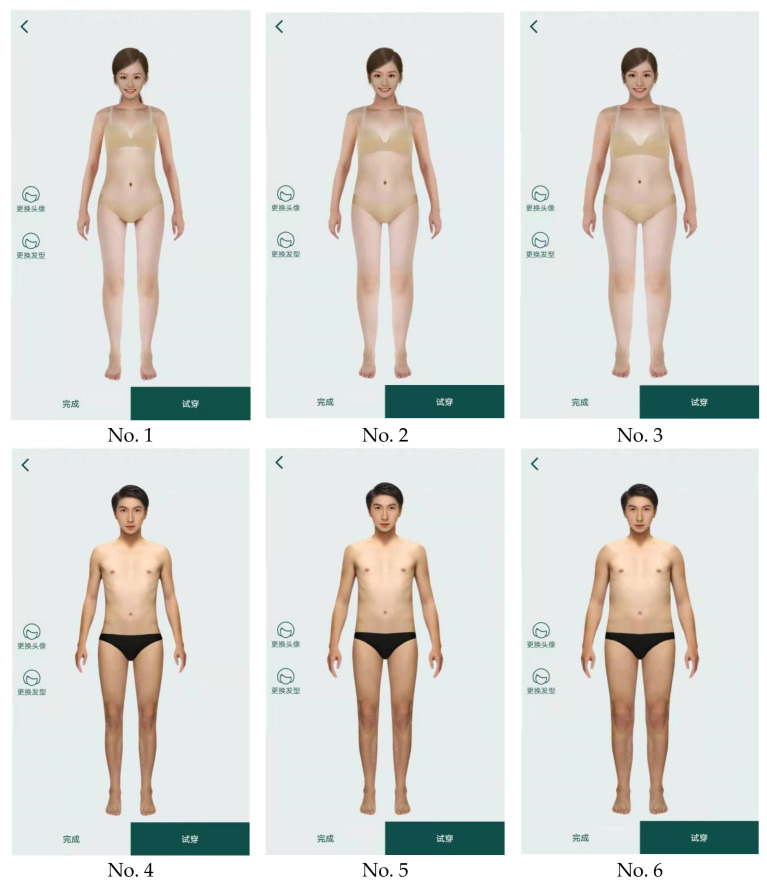
Six virtual models used in this study Note: the two emoticons in the figure imply changing the hair style, and the non-English term means “confirm or try-it on”.

## Data Availability

The dataset is available upon request from the authors.
